# Taurine regulation of short term synaptic plasticity in fragile X mice

**DOI:** 10.1186/1423-0127-17-S1-S15

**Published:** 2010-08-24

**Authors:** Abdeslem El Idrissi, Lorenz S Neuwirth, William L’Amoreaux

**Affiliations:** 1 Department of Biology, College of Staten Island, The City University of New York, 2800 Victory Boulevard, Staten Island, NY 10314, USA; 2 Doctoral Program in Biology – Neuroscience, The Graduate Center, The City University of New York, 365 Fifth Avenue, New York, NY 10016, USA; 3 Center for Developmental Neuroscience, College of Staten Island, The City University of New York, 2800 Victory Boulevard, Staten Island, NY 10314, USA; 4 Advanced Imaging Facility, College of Staten Island, The City University of New York, 2800 Victory Boulevard, Staten Island, NY 10314, USA

## Abstract

**Background:**

Fragile X Syndrome is the most common known genetic cause of autism. The *Fmr1*-KO mouse, lacks the fragile X mental retardation protein (FMRP), and is used as a model of the syndrome. The core behavioral deficits of autism may be conceptualized either as excessive adherence to patterns as seen in repetitive actions and aberrant language, or as insensitivity to subtle but socially important changes in patterns. The hippocampus receives information from the entorhinal cortex and plays a crucial role in the processing of patterned information. To gain more insight into the physiological function of FMRP and the neuronal mechanisms underlying fragile X syndrome, we examined the electrophysiological response of the hippocampus to pair pulse stimulation as a measure of patterned information processing and how it is affected in the *Fmr1*-KO mouse.

**Methods:**

In this study, we used paired-pulse stimulation of the afferent perforant path and recorded from the CA1 region of the hippocampus. Two-month-old FVB/NJ male mice and age-matched *Fmr1*-KO mice were used in this study. Hippocampal slices were prepared, equilibrated in artificial cerebrospinal fluid (aCSF), and excitatory post synaptic potentials (EPSPs) measured by stimulating the perforant path of the dentate gyrus (DG) while recording from the molecular layer of CA1. Stimulation occurred by setting current and pulse width to evoke a fixed percentage of maximal EPSP amplitude. This stimulation paradigm allowed us to examine the processing capabilities of the hippocampus as a function of increasing interstimulus intervals (ISI) and how taurine, a GABA_A_ receptor agonist, affects such information processing.

**Results:**

We found that hippocampal slices from wild type (WT) showed pair-pulse facilitation at ISI of 100-300 ms whereas slices from *Fmr1*-KO brains showed a consistent pair-pulse depression at a comparable ISI. Addition of 10 μM taurine to WT slices resulted in a drastic decrease of the peak response to the second stimulus, resulting in an initial depression at 100 ms ISI followed by potentiation at higher ISI (150 ms and above). In the presence of taurine, the amplitude of the second response remained significantly lower than in its absence. *Fmr1*-KO mice however, were completely insensitive to taurine application and pair-pulse stimulation always resulted in a depression of the response to the second stimulus.

**Conclusions:**

Previously we reported that *Fmr1*-KO mice have reduced beta subunits of the GABA_A_ receptors. We also showed as well as others that taurine acts as an agonist or a modulator for GABA_A_ receptors. Therefore, the insensitivity of *Fmr1*-KO slices to taurine application could be due to the reduced binding sites on the GABA_A_ receptors in the *Fmr1*-KO mice.

## Background

Fragile X is a common mental retardation syndrome that results from the silencing of a single gene, *FMR1*, on the X chromosome [1,2]. The loss of the *FMR1* gene product, FMRP, leads to mental retardation in males and to a range of deficits in heterozygous females. The syndrome has an autistic-like behavioral profile with hyper-arousal, self-stimulation, tactile-defensiveness and hypersensitivity to sensory stimuli [1,3]. There is also an increased prevalence of childhood seizures [4,5]. Physical features of the syndrome usually include enlarged testicles and prominent ears [4]. Neuroanatomical features include abnormal dendritic spine morphology [6-8], as well as alterations in the volume of the lateral ventricles and other structures [5,9]. FMRP is an RNA binding protein [10] that may act at the level of mRNA translation or translocation [11,12].

The fragile X mouse model for this disorder was generated by insertional mutagenesis of the mouse *Fmr1* gene that eliminated expression of the mouse protein, Fmrp [3]. The fragile X mouse exhibits features that resemble elements of the fragile X syndrome. These include enlarged testicles, mildly impaired learning behavior [3,13], an increased susceptibility to audiogenic seizures [14,15], altered behaviors associated with anxiety [16] and abnormal dendritic spine morphology [17-19]. The increase in susceptibility to seizures and spine dysgenesis may be due to the role of Fmrp as an activity-dependent regulator of translation [11,20,21]. Increased seizure susceptibility may be related to increased group 1 metabotropic receptor (mGluR) response [22] and to reduced GABA_A_ receptor expression [23]. In addition, long term depression (LTD) is enhanced in the fragile X mouse [24] and it has been proposed that this increase as well as spine dysgenesis are related to malfunction of a signaling cascade initiated by group 1 mGluRs in which Fmrp regulates translation of specific mRNAs [25].

Absence of Fmrp in the mouse model has also been shown to increase cerebellar LTD and to attenuate eye blink conditioning [26]. Analysis of the anterior piriform cortex showed reduced long term potentiation (LTP) in older animals [27]. No changes in paired pulse facilitation in hippocampal circuits have been noted when the interpulse interval was 40 ms [28] or 50 to 1600 ms [27]. However, these studies have examined the monosynaptic connections between the Schaffer collaterals and their postsynaptic target, the CA1 pyramidal cells. Here we have examined the integrative properties of the whole hippocampal formation and studied short-term plasticity within this structure and how it is affected by taurine.

## Methods

*Animals:* All mice used in this study were two-month-old FVB/NJ males. Mice were housed in groups of three in a pathogen-free room, maintained on a 12 hr light/dark cycle and given food and water ad libitum. All procedures were approved by the Institutional Animal Care and Use Committee of the College of Staten Island/CUNY, and were in conformity with the National Institutes of Health Guidelines. The number of mice sufficient to provide statistically reliable results was used in these studies.

*Slice preparation:* Hippocampal slices (400 µm) were prepared using an automated Leica tissue chopper. Artificial cerebrospinal fluid (aCSF) consisted of the following composition (mM): NaCl 124, KCl 3, CaCl_2_ 2.4, MgSO_4_ 1.3, NaH_2_PO_4_ 1.25, NaHCO_3_ 26, and glucose 10 (gassed with 95% O_2_ / 5% CO_2_, pH = 7.4). Slices were prepared on ice-cold aCSF and calibrated for 45min at room temperature (RT) in oxygenated aCSF prior to recording.

*Recording and stimulation:* Extracellular recordings of evoked excitatory postsynaptic potentials (EPSPs) were measured by placing the stimulating bipolar platinum electrodes in perforant path close to the suprapyamidal blade of the DG in the outer third of the molecular layer. Recording electrodes (tip impedance 1-5 MΩ) were placed in the middle or outer region of the molecular layer of CA1 to record EPSPs. This stimulation paradigm allowed us to assess both the evoked responses and the overall integrative properties of the whole hippocampus. Stimulation parameters for test pulses (0.05 Hz) were standardized across slices by setting current and pulse width to evoke a fixed percentage of maximal EPSP amplitude in any given data set. Similarly, stimulus intensity for plasticity induction was standardized for all data sets. The stimulus current that produced about 40% of maximal responses (usually 100– 150 μA) was used throughout the experiment for monitoring test pulses. Data were sampled and digitized at 100 kHz with a band-pass filtered between 0.3 Hz and 3 kHz.

*Statistical analysis:* Statistical significance was determined by Student's* t*-test. Each value was expressed as the mean ± SEM. Differences were considered statistically significant when the calculated *p* value was less than 0.05.

## Results

### Altered short term plasticity in the hippocampus of fragile X mice

**Figure 1:**
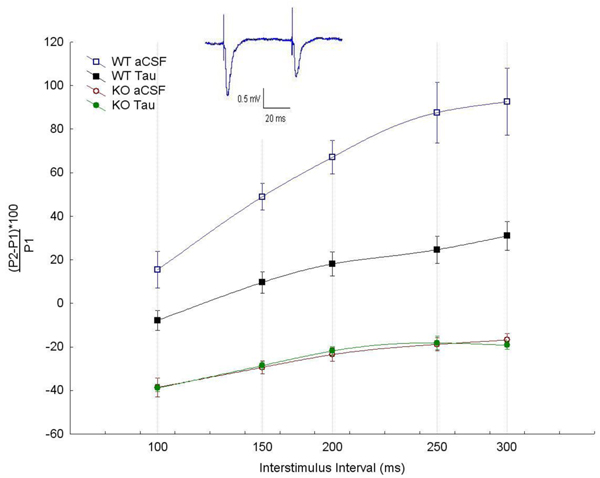
**Altered paired pulse facilitation in the fragile X hippocampus** Paired pulse stimulation at inter-stimulus intervals (ISI) of 100 ms and up to 300 ms consistently yielded facilitation as determined by the increase of the second EPSP (p2) compared to the first (p1). In Fmrp1-KO slices, stimulation of the hippocampal slices from Fmr1-KO resulted in a consistent depression of the second evoked response at all ISI used. Application of taurine (10µM) resulted in a paired pulse depression of WT responses but not Fmr1-KO. Each data point represents mean ± SEM of EPSP obtained following the paired pulse stimulation and expressed as [(p2-p1)/p1*100]. Data were obtained from 3 brains in each group and at least 5 slices per brain.

In the WT hippocampal slices, paired pulse stimulation at inter-stimulus intervals (ISI) of 100 ms and up to 300 ms consistently yielded facilitation as determined by the ratio of the evoked response to the second stimulus (p2) compared to the first stimulus (p1). On the other hand, stimulation of hippocampal slices from *Fmr1*-KO resulted in a consistent depression of the second evoked response at all ISI used (Figure 1). Although many factors could contribute to the observed electrophysiological differences between the WT and *Fmr1*-KO, alterations in the GABAergic system could be a contributing factor to these alterations in short term plasticity observed in the *Fmr1-*KO hippocampus. Consistent with this, bath application of taurine (10µM), a GABA_A_ receptor agonist, resulted in a paired pulse depression of WT responses but not *Fmr1*-KO (Figure 1).

## Discussion

We have previously shown that GABA_A_ receptor expression is down-regulated [23] and cortical GABAergic interneurons are less abundant in *Fmr1*-KO mice [29,30], suggesting reduced GABA signaling in these mice.

To assess electrophysiologically the functional significance of reduced expression of the GABA_A_ and the alterations of the inhibitory system in the *Fmr1*-KO hippocampus, we performed electrophysiological recording from the CA1 region of the hippocampus after paired pulse stimulation of the perforant path. This allowed us to determine the overall integrative properties of neuronal circuits within the hippocampal formation.

The finding that *Fmr1*-KO mice have altered paired pulse facilitation indicates that the mechanism underlying short-term plasticity in the hippocampus is compromised (Figure 1). Several factors could be at the origin of these alterations. We have shown that the GABAergic inhibitory system is altered in these mice [23]. Additionally, we showed that the expression of somatostatin is down-regulated in *Fmr1*-KO mice [30]. Furthermore we showed that the reduced expression of this neuropeptide is consistent with both central and peripheral features of the fragile X syndrome.

There are several studies indicating the interaction of taurine with GABA_A_ and glycine receptors acting as a modulator [31-36] or an agonist [37-44]. In heterologously expressed GABA_A_ receptors composed of either α1β3 or α1β3γ 2 subunits, taurine is a full or partial agonist, respectively, while both receptor types exhibit similar taurine sensitivities [44]. Interestingly we found that the *Fmr1*-KO hippocampal slices are irresponsive to bath application of taurine. While in the WT mice, taurine addition resulted in a significant decrease in the amplitude of the facilitation; in the *Fmr1*-KO, pair pulse stimulation resulted in a consistent depression of the second evoked response and addition of taurine had no effect. Taurine is a GABA_A_ receptor agonist and modulator [31-44]. Its application to hippocampal slices result in activation of GABA_A_ receptors and hyperpolarization of GABA_A_ receptors-expressing neurons, which comprises all principal cells of the hippocampus. This leads to reduced threshold for neuronal firing. Thus, under these conditions paired-pulse stimulation resulted, as expected, in a depression of neuronal response. The finding that slices from *Fmr1*-KO mice show abnormal pair pulse response and are insensitive to taurine indicates that the inhibitory GABAergic system is compromised in these mice and that the GABA_A_ receptors expressed in these mice lack the binding site for taurine. Consistent with this we showed that* Fmr1*-KO mice have reduced β subunit of the GABA_A_ receptors and taurine could presumably bind to this subunit (L’Amoreaux and El Idrissi, 2010; this volume). Furthermore, disrupting the gene for β3 subunit of the GABA_A_ receptor in mice produces electroencephalographic abnormalities, seizures, learning and memory deficits, poor motor skills on a repetitive task and hyperactivity features all common to fragile X syndrome [45]. Beta 3 subunit phosphorylation acts as a regulatory switch to control cycling of GABA_A_ receptors into and out of the plasma membrane [45]. This may allow neurons to specifically modify cell surface receptor insertion and/or endocytosis rates to control the efficacy of inhibitory synaptic transmission. The finding that* Fmr1*-KO mice have reduced β subunit of the GABA_A_ receptors may explain the abnormal response to pair pulse stimulation and the insensitivity to taurine application.

## Conclusions

*Fmr1*-KO mice have altered GABAergic system. Here, we show electrophysiologically that alterations in the GABAergic system may underlie the abnormal response to pair pulse stimulation, the cellular analogue of short-term plasticity. Furthermore, we showed that taurine regulates short-term plasticity in the hippocampus. Taurine application to slices resulted in a shift from pair pulse facilitation to pair pulse depression in WT but not *Fmr1*-KO mice presumably lacking the binding site for taurine on GABA_A_ receptors.

## Competing interests

The authors have no competing interests.

## Authors' contributions

AEI conceived of the study, designed the study, performed electrophysiological recordings, the statistical analysis and drafted the manuscript. LSN assisted in the dissections, recording and data processing. WJL participated in the study design as well as edited the manuscript. All authors read and approved the final manuscript.
